# Identification of Hazard and Socio-Demographic Patterns of Dengue Infections in a Colombian Subtropical Region from 2015 to 2020: Cox Regression Models and Statistical Analysis

**DOI:** 10.3390/tropicalmed8010030

**Published:** 2022-12-30

**Authors:** Santiago Ortiz, Alexandra Catano-Lopez, Henry Velasco, Juan P. Restrepo, Andrés Pérez-Coronado, Henry Laniado, Víctor Leiva

**Affiliations:** 1 School of Applied Sciences and Engineering, Universidad EAFIT, Medellín 050022, Colombia; 2 School of Industrial Engineering, Pontificia Universidad Católica de Valparaíso, Valparaíso 2362807, Chile

**Keywords:** arbovirus, clinical deterioration, endemic, proportional hazard, tropical diseases

## Abstract

Dengue is a disease of high interest for public health in the affected localities. Dengue virus is transmitted by *Aedes* species and presents hyperendemic behaviors in tropical and subtropical regions. Colombia is one of the countries most affected by the dengue virus in the Americas. Its central-west region is a hot spot in dengue transmission, especially the Department of Antioquia, which has suffered from multiple dengue outbreaks in recent years (2015–2016 and 2019–2020). In this article, we perform a retrospective analysis of the confirmed dengue cases in Antioquia, discriminating by both subregions and dengue severity from 2015 to 2020. First, we conduct an exploratory analysis of the epidemic data, and then a statistical survival analysis is carried out using a Cox regression model. Our findings allow the identification of the hazard and socio-demographic patterns of dengue infections in the Colombian subtropical region of Antioquia from 2015 to 2020.

## 1. Introduction

Dengue is a virus transmitted by *Aedes* species and distributed in tropical and subtropical regions [1]. Its main symptoms are fever, headache, and joint pain [2]. Diagnosing infected individuals in regions with the co-circulation of multiple arboviruses, such as zika and chikungunya, is challenging. In the case of dengue, it presents hyperendemic behaviors in tropical and subtropical regions, reporting approximately 96 million clinical cases per year [3]. Dengue is considered one of the most transmitted arboviruses in these regions [4], being endemic in some zones, for example, in the Americas [5].

Socio-economic conditions, changes in dengue over time, and the seasonal temperature variation induced by the El Niño Southern Oscitation (ENSO) influence the epidemiology of this infectious disease. Those aspects make the Americas a suitable place for epidemic outbreaks of dengue. Based on the reported cases, countries such as Brazil, Colombia, and Mexico are the most affected [6]. In the case of Colombia, the severe dengue fatality rate increased over time, as well as its incidence, due to the growing human population, the poor housing infrastructure, environmental drought/high rainfalls, and barriers to accessing health services [6,7].

Central-west Colombia is a dengue transmission hot spot, especially the Department of Antioquia, whose locality has the highest mortality and morbidity in dengue cases [8]. This subtropical locality has suffered from multiple outbreaks in recent years (2015–2016 and 2019–2020) [6,9,10,11]. Therefore, health authorities there have taken actions such as releasing mosquitoes with Wolbachia, controlling by fumigation, and cleaning breeding sites [12,13].

Dengue is considered a disease of high interest to public health. Several researchers have studied its behavior in both the Americas and Colombia specifically to understand the characteristics of infection and its appearance in the localities of interest [5,7,9,11]. For that reason, some studies implement survival analyses to identify the death or recovery time of a specific disease for vector-borne diseases [14,15,16]. However, there is still no evidence in the literature of sociodemographic or survival analyses of the impact of dengue on neglected localities in Colombia, such as the Department of Antioquia. Therefore, our main objective is to perform a retrospective analysis of the confirmed cases of dengue in Antioquia, discriminated by subregions and severity type from 2015 to 2020, through a descriptive analysis of the epidemic information and survival analysis using a Cox regression model.

We divide the manuscript as follows. In Section 2, we describe the implemented methodology with an overview of the case of study, the data management methods, the statistical analyses, and the implementation of the robust proportional hazard model. Section 3 reports the main results obtained for the Antioquia case study regarding both subregional behavior and dengue type. In Section 4, the discussion of our findings is presented. Finally, in Section 5, we present some conclusions of this research.

## 2. Methodology

### 2.1. Case Study

**Figure 1:**
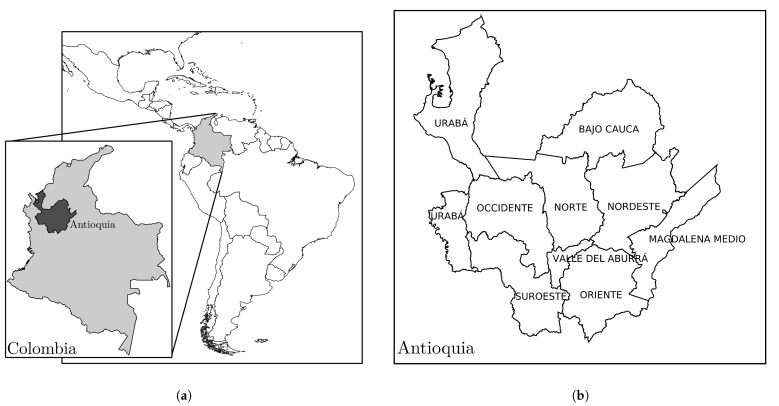
Study zone. (**a**) Location of Antioquia Department. (**b**) The territorial subdivision of Antioquia consists of nine subregions that have a high geographic diversity; see Table 1 for further information about each subregion.

Antioquia is a Department located in Northwest Colombia (Figure 1a) and has a population of around 6,550,206 inhabitants in an area of 63,612 km^2^. Its capital city is the municipality of Medellín (Table 1). Antioquia has entrances to the Caribbean Sea, which is crossed by the central and oriental cordillera. This geographical location generates heterogeneous environments and climates. The humid and semi-humid climates are found in the western, eastern, and central narrow strips. Its temperate and cold climates are distributed in the center of the department along the slopes of the central and western sides. Its precipitation ranges between 1500 mm and 4000 mm per year.

Regarding administrative divisions, Antioquia is distributed by nine subregions with their own environmental, geographical, and social characteristics (Figure 1b and Table 1 and Table 2), where 78.8% of its population live in urban zones and 21.2% in rural zones [19]. There are 149,535 registered immigrants, where the maximum number is located in Valle de Aburrá with 122,619, followed by Oriente with 13,458, and Urabá with 4489: nearly 1.9%, 0.2%, and 0.07% of these subregions’ populations, respectively. The remaining 8972 immigrants are distributed throughout other subregions [20].

The population in Antioquia is divided into the following age groups: early childhood (8.4%), childhood (8.6%), adolescence (10.8%), early adulthood (13.9%), adulthood (44.3%), and old age (14.0%) [20]. In addition, following the population pyramid developed by Gobernación de Antioquia [20], the subregions of Urabá, Magdalena Medio, Bajo Cauca, and Occidente present young populations with a high proportion of children and adolescents and a low number of adults and older adults.

In contrast, the subregions Este, Suroeste, Norte, and Nordeste have shown a decrease in children and adolescents and a gradual widening in the adult population. We highlight that Valle de Aburrá presents a decrease in the birth rate and life expectancy.

Concerning ethnic groups, Colombia recognizes four: (i) indigenous, (ii) Afro-Colombians (includes Afro-descendants, black people, mixed-race people, Palenqueros from San Basilio) (iii) Raizales from the San Andrés and Providencia archipelago, and (iv) ROM (Roma or Gypsy ethnicity). The indigenous groups live in their reservations or in the dispersed rural area of 32 municipalities; even so, this population has gradually mobilized from their territories to large urban centers, presenting a social issue. The ROM, Palenqueros, and Raizales populations are low in Antioquia, registering 140, 183, and 637 people, respectively. Most parts of these minorities are located in Valle de Aburrá and Urabá.

Depending on each subregion, there are localities with favorable conditions for the reproduction and feeding of *Aedes Aegypti*, having temperatures between 24 °C and 31 °C, humidity higher than 60%, and a high presence of breeding sites [25]. The first dengue hemorrhagic fever in Colombia was reported in Antioquia in 1989 [26]. Since then, a dengue alert has been in place throughout the country [8]. The ENSO enhances these arbovirus outbreaks through an increased vector population and feeding habits, as reported in other localities in South America [27,28]. Different outbreaks occurred in Colombia in 2010, 2015–2016, and 2019–2020 [10,11]. According to the Antioquia government, in 2016–2017, the most significant number of cases were in the Valle de Aburrá, Urabá, Occidente, and Suroeste localities. Some subregions could also provide under-registered arbovirus cases since many of them present favorable conditions for mosquito proliferation alongside precarious or non-existent health systems [9,29].

### 2.2. Dataset

The data to be used correspond to the daily cases of dengue in Antioquia, reported to the Sistema Nacional de Vigilancia en Salud Pública (SIVIGILA, Spanish acronym) during the years 2015–2020 (portalsivigila.ins.gov.co; accessed on 9 December 2020). The SIVIGILA is a system that associates users and procedures for collecting data to be analyzed and to obtain information from these data about health events in Colombia that must be interpreted and distributed. These data come directly from hospitals. The information is reported to the Instituto Nacional de Salud (INS, Spanish acronym), the institution in charge of public health surveillance in Colombia [10]. A case is considered a person who is suspected of infection and is reported to SIVIGILA. The arbovirus cases are classified into five groups: (i) suspect, (ii) probable, (iii) confirmed by the laboratory, (iv) confirmed by the clinic, and (v) confirmed by epidemiological nexus. The similarity between arbovirus symptoms can lead to misclassification biases on probable and suspect cases.

For correct analyses and to avoid misclassification issues, we discard unconfirmed cases, that is, the suspect and probable cases that are not confirmed by the laboratory, the clinic, or by epidemiological nexus [9,10]. Moreover, it is essential to point out that all individuals reported at SIVIGILA were alive patients, as this public institution is not responsible for registering and reporting deaths. We use public domain data collected by official government organizations, freely accessible and available by directly requesting them from these organizations. The health authorities anonymize these data related to social, economic, and symptomatological characteristics for each reported case. These data correspond to age, gender, subregion, municipality, area of occurrence (municipal seat or urban, rural, and dispersed rural), ethnic group (indigenous, ROM, Raizal, Palenquero, mixed-race, and Afro-Colombian), and occupation, according to the International Standard Classification of Occupations (ISCO-08) [30]. In addition, the hospitals report the event dates as epidemiological weeks, symptom onset, hospitalization, and death (the reported deaths correspond only to those directly registered in the SIVIGILA). The cleaned data set and the corresponding computational code in the R statistical software [31] are available at github.com/alexacl95/DengueAntioquia (accessed on 11 July 2022).

### 2.3. Statistical Analyses

We consider quantitative and qualitative variables according to both subregion location and by type of dengue: common or typical dengue and severe dengue. Subregions only consider socio-demographic variables. For subregional analysis, all quantitative variables are expressed in terms of their sample median, along with their respective 95% bootstrap confidence intervals. For multiple testing, such medians are compared using the Kruskal–Wallis (KW) test. If sample evidence rejects the null hypothesis that the variable differs by subregion, based on the KW test, then all the subregions are compared using a two-sided pairwise Mann–Whitney (MW) test with Holm–Bonferronni correction. Qualitative variables are expressed in their absolute/relative frequencies and compared with subregions by the independence chi-squared test with Yate correction for small samples, if needed.

For comparative analysis by dengue type, quantitative variables are expressed again in terms of their sample median and their respective 95% bootstrap confidence intervals and compared using a two-sided MW test. Similarly, qualitative variables are expressed in their absolute/relative frequencies and compared using a two-sided, two-sample Z test for proportions. We consider the robust proportional hazard model [32], a robust version of the well-known Cox regression [33], to identify covariates that can explain the hazard ratio, avoiding possible interference of outlying observations. This model is formulated for the dependent variable “clinical deterioration time”, measured in days, which expresses the delay from symptoms onset to hospital admission. Here, we are interested in measuring the impact of socio-demographic variables on the probability that an infected person requires more days to deteriorate. We perform all statistical analysis in the R software. Specifically the coxrobust package [34] is used to formulate the robust Cox regression model.

## 3. Results

### 3.1. Dengue in Antioquia: 2015–2020

**Figure 2:**
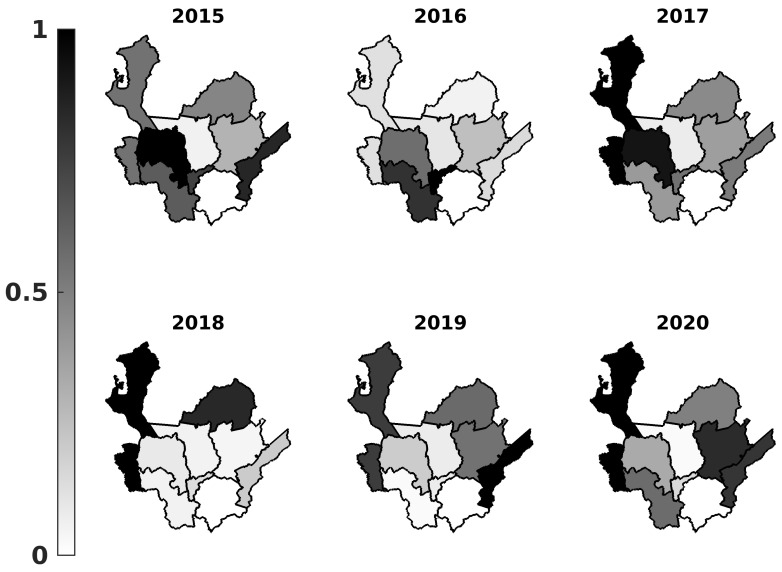
Choropleth map of normalized dengue prevalence in Colombia for cases reported during 2015–2020.

In total, 50,397 people with dengue infection were reported, of which only 491 (0.97%) could not be georeferenced. About 80% of the total infections occurred in two subregions, with the majority of cases being located in Valle de Aburrá with 35,335 (70.1%), followed by Urabá with 5196 cases (10.3%). However, the relevant incidence was in Urabá (2017, 2018, and 2020), followed by Magdalena Medio (2015 and 2019), Occidente (2015 and 2017), and Bajo Cauca (2018); see Table 3 and Figure 2. Those subregions are characterized as localities with high temperatures and low altitudes (Table 1): favorable environmental conditions for the reproduction of *Aedes* species.

**Figure 3:**
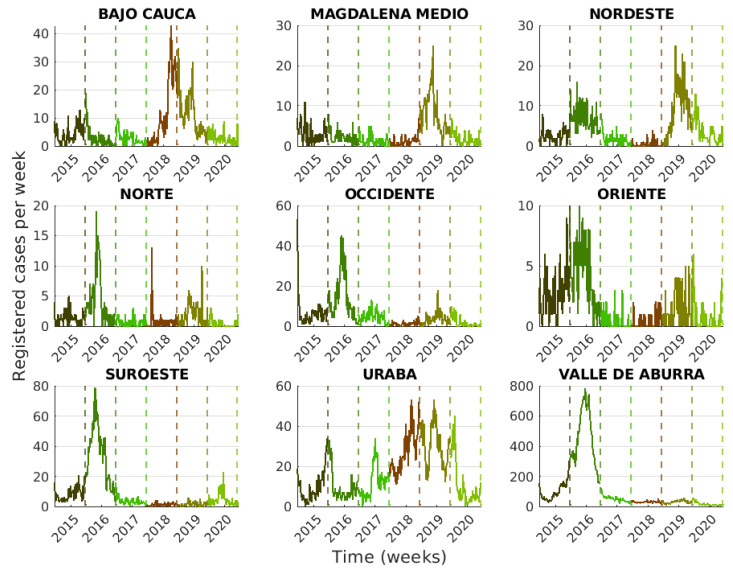
Time-series of reported dengue cases per subregion during 2015–2020. Different colors in the graphs represent the time-frames.

In Figure 2 and Figure 3, we present the number of people affected by dengue over the total population (normalized prevalence per subregion) and the registered dengue cases as time series. A prevalence map is presented using the number of cases per year and the inhabitants per subregion (Table 1), where prevalence in each year was normalized to be between 0 and 1; see Table 3 for the exact values. By normalizing the prevalence values, we can visualize the most affected localities per year. Figure 3 shows the dengue cases’ time series per subregion and year corresponding to Southern localities (Suroeste, Occidente, and Valle de Aburra) and Northern localities (Uraba, Bajo Cauca, and Magdalena), which presented outbreaks in 2015–2016 and 2018–2020, respectively. According to Figure 2 and Figure 3, the epidemic outbreaks occurred in different periods for some localities. Suroeste, Occidente, Oriente, and Valle de Aburrá presented outbreaks during 2015–2016, whereas Bajo Cauca, Nordeste, and Magdalena Medio saw increased numbers of endemic cases. The 2015–2016 period corresponds to one of the most intense ENSO phenomena reported in the last years. In addition, Northern localities such as Urabá, Bajo Cauca, Nordeste, and Magdalena Medio suffered dengue outbreaks during 2018–2020 and reported a normal/moderate ENSO effect over the region during this period.

**Figure 4:**
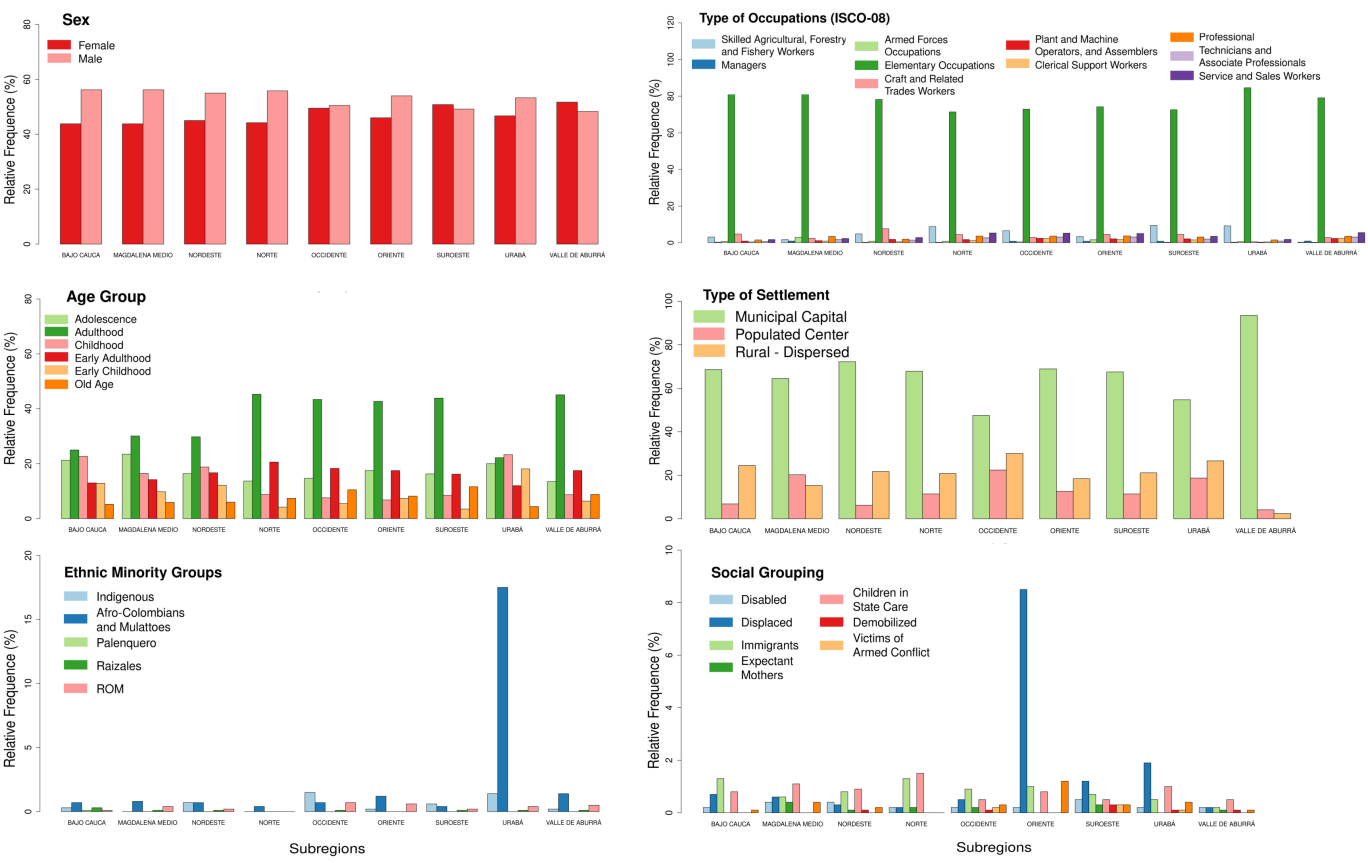
Barcharts for socio-demographic variables, discriminated by subregion, including sex, age groups, ethnic minority groups, type of occupation, and social grouping. For more details and *p*-values, we refer the reader to Table 4 and Table 5.

For all socio-demographic variables by subregions, statistical significance was detected using the chi-squared test (Table 4 and Table 5). The results indicate that a relationship exists between socio-demographic variables and subregions. Figure 4 summarizes Table 4 and Table 5, where we note that men suffer from more dengue infections than women, except in Suroeste and Valle de Aburrá. In addition, people in the adult age group had more infections than other age groups. Nevertheless, in Urabá and Bajo Cauca, younger people (adolescents and children) have a similar prevalence as adults. Regarding the remaining socio-demographic variables, the most affected people, around 80%, were those whose principal occupation was elementary, such as cleaners, agriculture workers, food preparers, mining/construction workers, or street sales workers: occupations that were highly correlated to people with medium-low socioeconomic status. Regarding the location, more than 40% of cases occurred in a municipal capital for all subregions. Hence, according to ethnic minority groups, Afro-Colombians and mixed-race people were the most affected groups in all subregions, especially in Urabá, but in Suroeste and Occidente, indigenous people were the most affected (Figure 4).

### 3.2. Symptomatological Behavior by Both Subregion and by Type of Dengue

By subregion, all medical and symptomatological variables were significant according to the KW and chi-squared tests. The “medical consultation time” (in days) is when a person starts having symptoms and consults in a medical center. The “clinical deterioration time” (in days) is the time between when a person enters the center and when they are transferred to hospitalization (Table 6).

According to the MW test *p*-values (Table 7), there was a statistically significant difference between the subregions in terms of medical consultation time, except for the following pairs: Bajo Cauca–Oriente, Suroeste–Magdalena Medio, Nordeste–Occidente, Norte–Urabá, Norte–Valle de Aburrá, and Suroeste–Oriente. Moreover, there were statistically significant differences in clinical deterioration time between the subregions Suroeste–Bajo Cauca, Valle de Aburrá–Occidente, and Magadalena Medio and the rest (except Occidente and Valle de Aburrá–Oriente). Similar symptoms occurred in all subregions.

People suffered from common dengue symptoms, and the proportions by subregion were close to each other. Relevant findings were the rate of hospitalization and severe dengue events, with Urabá and Bajo Cauca having the highest percentages of hospitalized patients, for either normal or severe dengue, with 57.7% and 48.4%, respectively. In contrast, Suroeste and Valle de Aburrá had the smallest percentages of severe dengue cases (0.4%), about half compared to the other subregions (Table 6). Interestingly, Valle de Aburrá is the most populated subregion of Antioquia, within which the city of Medellín is located.

According to the type of dengue (Table 8), there were 50,101 people with dengue (99.4%) and 296 people with severe dengue (0.6%). The percentage of men and women with dengue and severe dengue was the same. The number of adults with dengue was greater than adults with severe dengue (41.3% versus 29.7%, *p*-value < 0.0001). Furthermore, the number of older people with dengue was less than that of older people with severe dengue (8.2% versus 11.8%, *p*-value = 0.03). There were no statistical differences in the other age group categories in both dengue-type groups.

The medical consultation time of people with dengue was statistically less than people with severe dengue (four days versus three days, *p*-value < 0.0001), even considering the median bootstrap confidence intervals. Nonetheless, the clinical deterioration time between the two groups was not statistically different. The greatest differences in both groups were in the hospitalization requirement. More people with severe dengue were hospitalized compared to those with normal dengue (98% versus 29.3%, *p*-value < 0.0001). Regarding symptomatology, fever, retro-ocular pain, myalgia, and arthralgia were not statistically different between the dengue and severe dengue groups. Moreover, headache (86.1% versus 79.7%, *p*-value = 0.002) and rash (48.1% versus 38.5%, *p*-value = 0.001) were statistically different symptoms whose frequency was greater in normal dengue patients than in severe dengue patients (Table 8).

For the remaining symptoms, that is, abdominal pain (26% versus 73.6%), vomiting (22.8% versus 54.4%), diarrhea (15% versus 32.4%), drowsiness (3.2% versus 22%), hypotension (1.5% versus 28%), hepatomegaly (1.2% versus 13.5%), oral ecchymosis (3.6% versus 19.6%), hypothermia (0.4% versus 6.8%), thrombocytopenia (21.5% versus 73%), and high hematocrits levels (3.1% versus 23.6%), there were statistically significant differences between both dengue groups, all these comparisons having a *p*-value < 0.0001 (Table 8).

It is essential to point out that these last symptoms were more frequent in the severe dengue group than in the normal one, achieving values at least twice larger than in the normal group. Thus, it would be expected that patients presenting these symptoms are likely related to future severe dengue conditions.

### 3.3. Impact of Socio-Demographic Variables in Clinical Deterioration Time of Hospitalized Patients

A total of 14,960 people were hospitalized (Table 8). We formulate a robust Cox regression to model the impact of some socio-demographic variables on the hazard rate function of the response variable “clinical deterioration time”. In this model, the covariates were: sex (male/female), type of dengue (normal/severe), type of settlement (municipal capital/populated center/rural-dispersed), and subregion (all nine).

In a previous descriptive analysis, we found that the behavior of the variable “clinical deterioration time” was common among age groups. In addition, the first and third quartiles as well as the median values were very similar (3, 6, and 4, respectively). Therefore, we decided not to adjust the Cox regression on age. Furthermore, the inclusion of more binary variables in this regression could affect the estimates due to the decrease in the degrees of freedom.

**Figure 5:**
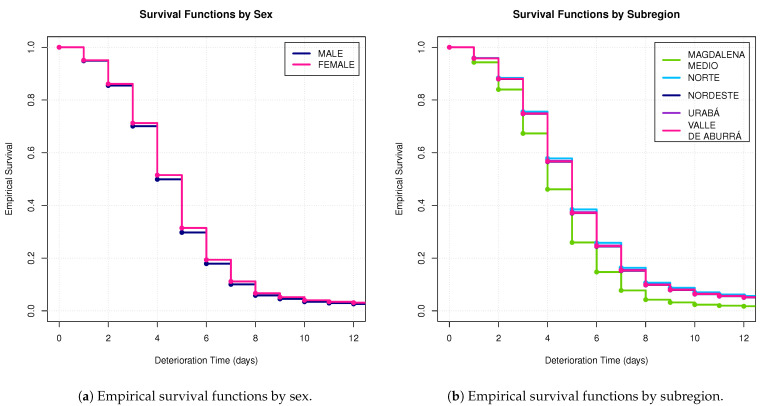
Survival functions for deterioration time according to the robust Cox regression model presented in Table 9.

The baseline levels for the binary variables sex and type of dengue were female and normal, respectively. Regarding the remaining multilevel variables, we structured them in a binary form. In Table 9, we summarize this information, where the target levels are presented in brackets. According to the robust proportional hazard model, only the type of dengue variable was not statistically significant (*p*-value = 0.139) for describing the hazard rate (Table 9). This implies that the type of dengue does not influence the probability that a person will require more days to deteriorate and be admitted to the hospital. The sex variable was significant (*p*-value = 0.013) with an estimated regression coefficient of 0.047, which shows that, considering the other variables as fixed, men have an impact of 1.048 in the estimated hazard rate; that is, male patients are more likely to have a faster clinical deterioration than women. Therefore, women require more days to show clinical deterioration than men (Figure 5a).

Regarding the variable type of settlement, it was statistically significant for the binary relation “populated center–not populated center” (*p*-value = 0.001), with an estimated regression coefficient of 0.120, indicating that, fixing the other variables, people that live in a populated center have an impact of 1.127 in the estimated hazard rate; that is, people in populated centers are more likely to have a faster clinical deterioration compared to people that do not live in a populated center (Figure 5b). Moreover, for the subregion variable, five binary relations were statistically significant (Table 9). The relation “Magdalena Medio–not Magdalena Medio” was the only one with a positive estimated coefficient (0.154). Thus, people living in a Magdalena Medio have the highest probability of instant clinical deterioration compared to who do not live in that area. The whole comparison between subregions, in terms of the estimated survival functions, is shown in Figure 5b.

## 4. Discussion

### 4.1. Dengue in Colombia

Dengue virus is one of the most important arboviral infections worldwide because of its incidence in tropical and subtropical regions [2]. Among these regions, Colombia is one of the most important, as it presents multiple epidemics and hotspot zones. Thus, any social, epidemiological, and medical information about the incidence of the dengue virus in Colombia is crucial in future research for understanding and designing control policies, considering that Colombia has the highest medical cost per day, followed by Vietnam and Thailand [35]. In this research, we presented a retrospective study of dengue’s impact on one of Colombia’s most affected zones from 2015 to 2020, the Department of Antioquia.

Regarding how relevant it is to contrast regions systematically, note that, in Colombia, the department of Antioquia is the most critical region in terms of dengue incidence. To the best of our knowledge, detailed analyses of a specific area at the regional and subregional levels have not been carried out so far. Therefore, we consider it an important issue to study this phenomenon at the subregional level due to the potential impact it can generate.

### 4.2. Socio-Economic State of Dengue in Antioquia

Different factors affect the propagation of the dengue virus over a susceptible population. These are related to health conditions, access to essential services, and socio-economic conditions, which in turn are related to the vector life cycle and proliferation [7]. The assumption of constant mortality and birth rates of mosquitoes is not suitable. This is because they vary depending on environmental conditions [36,37] and variations in both temperature and rain levels, which could increase or decrease these rates [38], as well as affect the bite rate and incubation periods of mosquito offspring [39]. For Antioquia, some subregions that reported outbreaks during 2015–2016 were experiencing one of the most intense ENSO phenomena reported in last years [40], and the localities affected during 2018–2020 were experiencing a moderate ENSO period [41].

Arbovirus transmission mainly comes from urban areas with high population density, medium-low economic class, and poor infrastructure [42] that facilitates *Aedes* breeding sites, for example, water supplies or sewage systems [7]. As pointed out in [43,44,45], socio-economic factors such as proximity to stagnant waters, poverty, invasions, localized violence, and military migration are some statistically significant risk factors that contribute to a high endemicity. According to our findings, Urabá, Bajo Cauca, and Magdalena Medio had the highest values of dengue prevalence (greater than 50%) over six years (Figure 2). These first two subregions are characterized by having high poverty, overcrowding, and misery rates, as discussed next.

Further information is provided in Table 10 related to urban, rural, and total population indices. This table reports the percentage of people or households that belong to the following specific categories:

(i) Poverty indicates the percentage of people that cannot pay for essential resources.
(ii) The health barrier shows the percentage of individuals or families that cannot access health services in hospitals.
(iii) No access to water measures the percentage of households with no access to an adequate water supply, such as potable water.
(iv) Overcrowding measures homes with over three people per room, counting the living rooms and dining room but excluding bathrooms, garages, and rooms used for businesses.

In Antioquia, the most affected populations were:

(i) Adulthood, a working age that represents 44.3% of the total population [20].
(ii) People in elementary occupations (all subregions).
(iii) Displaced, a minority representing 1.1% of the total population in Antioquia; in addition, the term displaced is associated with victims of the Colombian armed conflict, another population representing almost 20% of the population of the whole department [20,21].
(iv) Afro-Colombians in Oriente and Urabá; the last region is this community’s major settlement, and 36% of its population lives in rural zones [46].
(v) Immigrant groups, where 81% of the population is made up of people from Venezuela, followed by people from the United States and Ecuador [46].
(vi) Children in state care (all subregions).

These communities are vulnerable populations exposed to precarious conditions and forced migrations from other precarious localities. There is a positive relationship between prevalence, land ownership, migration, and forced displacement. This is especially detected in Urabá and Oriente, the most important agricultural production subregions [47], which have been strongly affected by the events of the Colombian armed conflict [48].

Regarding dengue vaccination, currently, there are some licensed vaccines, for example, CYD-TDV, Dengvaxia, Sanofi Pasteur, and candidates such as TAK-003 [49,50]. The last one has been clinically tried in Colombia and presents high efficiency for DENV-2, but not for other serotypes [49]. The idea of developing immunization campaigns for vulnerable populations is closer to being realized in Colombia. Even so, it is important to point out that Colombia is a hyperendemic country in which the predominant serotype may change [39]. Thus, the vaccination process must consider serotype co-circulation analyses to guarantee proficient immunization.

### 4.3. Socio-Demographic Hazards and Relationship of Dengue and Severe Dengue Symptoms

At a general level in Antioquia, we identified no significant difference between men and women in the prevalence of dengue and severe dengue (Table 8). However, the sex prevalence changes at the subregional level. For example, in Bajo Cauca, Magdalena Medio, and Nordeste, a high male prevalence was observed; see Table 2 and Table 4 for the sex ratio per subregion. In [51], it was reported that severe dengue is mostly female-biased after puberty and unbiased for the rest of the age classes. Thus, there is no unique pattern for other localities in the world. For example, Singapore reported no sex bias [52], while Pakistan [53] has a high prevalence for men, and Brazil [54] and Nicaragua [55] have higher prevalences for females. We detected that working ages in Antioquia (adults between 27 and 86 years old) are the most affected group, similar to the results reported in some localities in Brazil [54], Pakistan [56], and Saudi Arabia [57], where the average age of affected adults ranges between 22 and 52 years [54]. We could explain this trend from the socio-economical approach: working ages are high-mobility groups that travel outside their neighborhood, spending over seven hours per day in educational institutions or working areas. This dynamic increases the probability of dengue, especially in high-mosquito-density areas [57].

We assessed that hemorrhagic manifestations are also adult-biased in Antioquia, where approximately 60% of the severe cases are adults above 18 years, as reported in [53,54]. Primary infection is a protective factor against the hemorrhagic forms in children since repeated infections by a different serotype of the dengue result in more severe manifestations [51,54]. The most common symptoms in Antioquia are almost the same: fever, headache, retro-ocular pain, myalgia, arthralgia, and rash, as reported in [53,54]. The hospitalization rate in Antioquia is 29.6% of the cases over the last five years, with Bajo Cauca and Urabá being the most affected subregions. Other studies reported a lower hospitalization, for example, 13.2% of the reported cases [54].

The main results of survival analysis with the robust Cox regression showed that the factors that affect the probability of instant clinical deterioration are sex and location. According to our findings, men suffer from a faster clinical deterioration than women. In addition, people that live in crowded areas, such as Magdalena Medio, mainly characterized by high poverty levels, bad sanitary conditions, informal employment (elementary occupations in general), and vulnerable populations (displaced, immigrants, or children in state care), suffer a faster clinical deterioration. This is an important result on the topic as survival analyses in dengue have been principally implemented for other studies, such as the rate of healing on severe dengue patients [58,59] and survival rates [56].

### 4.4. Dengue Infections with the COVID-19 Pandemic

The year 2020 was remarkable for the study of epidemiology. The control of COVID-19’s propagation affected the incidence, treatment, and identification of other infectious diseases.

After the effects of COVID-19 on the total population, the World Health Organization reported over 1.6 million arboviral cases in the Americas, with the majority (about 97%) being dengue. At the end of 2019 and the beginning of 2020, a dengue outbreak started in Colombia [11]. Its prevalence was less than that reported in the same period in 2019 [60,61]. Some subregions of Antioquia, such as Magdalena, Nordeste, Bajo Cauca, Urabá, and Occidente, presented a decrease in incidence; see Table 3 and Figure 3. Some recent studies relate the decrease in dengue cases to the effect of COVID-19 on health facilities and the diagnosis becoming problematic because both diseases exhibit similar clinical and laboratory manifestations [62]. Other authors attribute the decrease in dengue cases to the lockdowns and declines in regional migration [61]. In [63], a strong association was highlighted between COVID-19-related social changes and the reduction in dengue transmission (school closures and reduced time spent in nonresidential areas). This finding shows evidence that dengue transmission occurred in shared areas outside the home [61].

## 5. Conclusions

Dengue virus is transmitted by *Aedes* species and presents hyperendemic behaviors in tropical-subtropical regions. Colombia is one of the most affected countries in the Americas. The central-west region is a hot spot in dengue transmission, especially the subtropical localities of the Department of Antioquia. This zone has suffered multiple dengue outbreaks recently (2015–2016 and 2019–2020). As dengue is a disease of high interest to public health in the affected localities, we have formulated Cox regression models and conducted statistical analyses to identify the hazard and socio-demographic patterns of this infectious disease in a Colombian subtropical region between the years of 2015 and 2020. Hence, we performed a retrospective analysis of the confirmed dengue cases in Antioquia by discriminating by both subregions and dengue severity during these years. First, we conducted an exploratory analysis of the epidemic data, and then we formulated a statistical survival analysis using a Cox regression model. Our findings allowed the identification of the hazard and socio-demographic patterns of dengue infections in Antioquia, Colombia, from 2015 to 2020.

We studied the clinical deterioration time. A possible future work might be related to a survival analysis for the time to medical consultation. A comparison of our results with other similar works in the South American region, including Andean countries, is proposed for future research. This will allow us to have a global vision of Dengue and not a local country vision, given the importance of this disease in the Americas as endemic.

## Figures and Tables

**Table 1:** Summary of geographical and climatic characteristics for the nine subregions in Antioquia. For further information about description of each subregion, see [17,18].

Subregion	Location	Area (km^2^)	Altitude (MSL)	Temperature Range (°C)
Valle de Aburrá	South center	1158	1300–1775	12–21
Bajo Cauca	Northeast, in the spur of the CC	8585	30–125	17–29
Norte	North, in CC	7516	1200–2550	12–23
Nordeste	Eastern slopes of the CC	8645	650–1975	19–27
Suroeste	Southwestern, between WC and CC	6589	600–2350	12–26
Occidente	Northwest, between WC and CC	6571	450–1925	10–26
Oriente	Southeast	7103	1000–2500	13–23
Urabá	North, Coastal region	11,799	2–200	22–29
Magdalena Medio	CC	4833	75–950	24–29

Where central cordillera (CC) and west cordillera (WC).

**Table 2:** Distribution of the population that corresponds to the demographic groups in each subregion [20,21,22,23,24]. * There are 1,307,620 victims reported in Antioquia (nearly 20% of the population); even so, 704,811 of them are not georeferenced [21].

Subregion	Population	Gender Men	Social Groups			Settlement (Urban)	Minorities	
			Disabled	Displaced	Victims *		Indigenous	Mixed-Race and Afro-Colombian
Valle de Aburrá	3,969,222	53%	2.1%	0.07%	2%	97%	0.1%	1.9%
Bajo Cauca	255,064	50%	1.7%	2.21%	21%	65%	2.3%	6.9%
Norte	244,995	51%	3.1%	1.61%	19%	50%	0.22%	1.26%
Nordeste	199,335	50%	2.9%	1.17%	16%	54%	0.46%	0.91%
Suroeste	367,467	50%	3.1%	0.84%	14%	48%	1.22%	0.75%
Occidente	210,371	51%	3.3%	3.65%	24%	38%	4.15%	1.50%
Oriente	683,968	49%	2.6%	2.34%	18%	60%	0.05%	0.37%
Urabá	514,423	49%	1.8%	5.22%	28%	59%	2.67%	39%
Magdalena Medio	105,361	51%	3.7%	2.38%	9%	56%	0.11%	2.55%

**Table 3:** Dengue incidence per 1000 inhabitants by subregions between the years 2015 and 2020.

Subregion	Year						Median Incidence
	2015	2016	2017	2018	2019	2020	
BC	0.90	0.61	0.57	2.32	2.39	0.55	0.755
MM	1.44	1.11	0.64	0.59	4.00	0.84	0.975
NE	0.64	1.73	0.48	0.13	2.26	0.88	0.760
NO	0.28	0.88	0.12	0.17	0.40	0.08	0.225
OC	1.66	3.51	1.11	0.27	0.88	0.39	0.995
OR	0.20	0.32	0.04	0.03	0.11	0.06	0.085
SO	1.14	4.84	0.50	0.17	0.19	0.63	0.565
UR	1.01	1.01	1.20	2.75	3.08	1.05	1.125
VA	1.29	5.94	0.68	0.38	0.39	0.21	0.535
Median Incidence	1.01	1.11	0.57	0.27	0.88	0.55	0.725

Where Bajo Cauca (BC), Magdalena Medio (MM), Nordeste (NE), Norte (NO), Occidente (OC), Oriente (OR), Suroeste (SO), Urabá (UR), and Valle de Aburrá (VA).

**Table 4:** Socio-demographic characteristics among patients, according to the subregion.

Variable		BC (n = 1874)	MM (n = 908)	NE (n = 1218)	NO (n = 475)	OC (n = 1644)	OR (n = 515)	NG (n = 491)	SO (n = 2741)	UR (n = 5196)	VA (n = 35,335)	*p*-Value
	Age	16 (15–16.5)	19 (17-20)	19 (18–21)	28 (26-30)	28.5 (27–29.5)	27 (25–29)	28 (26–31)	30 (29–31)	14 (14–15)	28 (28–29)	<0.0001
Age group	Early childhood (0–5)	241 (12.9%)	89 (9.8%)	149 (12.2%)	20 (4.2%)	90 (5.5%)	38 (7.4%)	37 (7.5%)	95 (3.5%)	943 (18.1%)	2251 (6.4%)	<0.0001
	Childhood (6–11)	426 (22.7%)	150 (16.5%)	229 (18.8%)	42 (8.8%)	125 (7.6%)	35 (6.8%)	46 (9.4%)	234 (8.5%)	1209 (23.3%)	3085 (8.7%)	
	Adolescence (12–18)	399 (21.3%)	213 (23.5%)	200 (16.4%)	65 (13.7%)	241 (14.7%)	90 (17.5%)	62 (12.6%)	446 (16.3%)	1039 (20%)	4773 (13.5%)	
	Early adulthood (19–26)	243 (13%)	129 (14.2%)	204 (16.7%)	98 (20.6%)	301 (18.3%)	90 (17.5%)	85 (17.3%)	444 (16.2%)	622 (12%)	6196 (17.5%)	
	Adulthood (27–59)	468 (25%)	273 (30.1%)	363 (29.8%)	215 (45.3%)	714 (43.4%)	220 (42.7%)	214 (43.6%)	1204 (43.9%)	1154 (22.2%)	15932 (45.1%)	
	Old age (60+)	97 (5.2%)	54 (5.9%)	73 (6%)	35 (7.4%)	173 (10.5%)	42 (8.2%)	47 (9.6%)	318 (11.6%)	229 (4.4%)	3098 (8.8%)	
Sex	Female	820 (43.8%)	398 (43.8%)	548 (45%)	210 (44.2%)	814 (49.5%)	237 (46%)	235 (47.9%)	1392 (50.8%)	2429 (46.7%)	18256 (51.7%)	<0.0001
	Male	1054 (56.2%)	510 (56.2%)	670 (55%)	265 (55.8%)	830 (50.5%)	278 (54%)	256 (52.1%)	1349 (49.2%)	2767 (53.3%)	17079 (48.3%)	
Type of settlement	Municipal capital	1286 (68.6%)	586 (64.5%)	879 (72.2%)	322 (67.8%)	781 (47.5%)	355 (68.9%)	442 (90%)	1849 (67.5%)	2844 (54.7%)	33055 (93.5%)	<0.0001
	Populated center	128 (6.8%)	183 (20.2%)	75 (6.2%)	54 (11.4%)	369 (22.4%)	65 (12.6%)	19 (3.9%)	313 (11.4%)	970 (18.7%)	1443 (4.1%)	
	Rural–dispersed	460 (24.5%)	139 (15.3%)	264 (21.7%)	99 (20.8%)	494 (30%)	95 (18.4%)	30 (6.1%)	579 (21.1%)	1382 (26.6%)	837 (2.4%)	
Type of occupation (ISCO-08)	Skilled agricultural, forestry, and fishery workers	460 (24.5%)	139 (15.3%)	264 (21.7%)	99 (20.8%)	494 (30%)	95 (18.4%)	30 (6.1%)	579 (21.1%)	1382 (26.6%)	837 (2.4%)	<0.0001
	Managers	5 (0.3%)	8 (0.9%)	2 (0.2%)	1 (0.2%)	13 (0.8%)	4 (0.8%)	9 (1.8%)	23 (0.8%)	14 (0.3%)	328 (0.9%)	
	Armed forces	13 (0.7%)	27 (3%)	10 (0.8%)	4 (0.8%)	6 (0.4%)	8 (1.6%)	11 (2.2%)	6 (0.2%)	29 (0.6%)	63 (0.2%)	
	Elementary occupations	1612 (86%)	751 (82.7%)	953 (78.2%)	339 (71.4%)	1198 (72.9%)	382 (74.2%)	340 (69.2%)	1989 (72.6%)	4398 (84.6%)	27950 (79.1%)	
	Craft and related trades workers	88 (4.7%)	22 (2.4%)	93 (7.6%)	21 (4.4%)	48 (2.9%)	23 (4.5%)	15 (3.1%)	124 (4.5%)	25 (0.5%)	993 (2.8%)	
	Plant and machine operators and assemblers	17 (0.9%)	10 (1.1%)	22 (1.8%)	8 (1.7%)	39 (2.4%)	11 (2.1%)	11 (2.2%)	58 (2.1%)	14 (0.3%)	800 (2.3%)	
	Clerical support workers	8 (0.4%)	8 (0.9%)	6 (0.5%)	5 (1.1%)	37 (2.3%)	9 (1.7%)	9 (1.8%)	42 (1.5%)	21 (0.4%)	840 (2.4%)	
	Professionals	29 (1.5%)	31 (3.4%)	23 (1.9%)	17 (3.6%)	60 (3.6%)	19 (3.7%)	38 (7.7%)	86 (3.1%)	76 (1.5%)	1221 (3.5%)	
	Technicians and associate professionals	12 (0.6%)	15 (1.7%)	17 (1.4%)	13 (2.7%)	51 (3.1%)	16 (3.1%)	30 (6.1%)	56 (2%)	47 (0.9%)	1107 (3.1%)	
	Service and sales workers	31 (1.7%)	21 (2.3%)	34 (2.8%)	25 (5.3%)	85 (5.2%)	26 (5%)	21 (4.3%)	97 (3.5%)	93 (1.8%)	1940 (5.5%)	

Where Bajo Cauca (BC), Magdalena Medio (MM), Nordeste (NE), Norte (NO), Occidente (OC), Oriente (OR), not georeferenced (NG), Suroeste (SO), Urabá (UR), and Valle de Aburrá (VA).

**Table 5:** Socio-demographic characteristics among patients, according to subregion location (part B).

Variable		BC (n = 1874)	MM (n = 908)	NE (n = 1218)	NO (n = 475)	OC (n = 1644)	OR (n = 515)	NG (n = 491)	SO (n = 2741)	UR (n = 5196)	VA (n = 35,335)	*p*-Value
Ethnic minority groups	Indigenous	5 (0.3%)	0 (0%)	9 (0.7%)	0 (0%)	24 (1.5%)	1 (0.2%)	2 (0.4%)	16 (0.6%)	72 (1.4%)	66 (0.2%)	<0.0001
	Afro-Colombians and mixed-race	13 (0.7%)	7 (0.8%)	9 (0.7%)	2 (0.4%)	11 (0.7%)	6 (1.2%)	6 (1.2%)	12 (0.4%)	907 (17.5%)	496 (1.4%)	
	Palenquero	1 (0.1%)	0 (0%)	0 (0%)	0 (0%)	0 (0%)	0 (0%)	0 (0%)	0 (0%)	1 (0%)	2 (0%)	
	Raizales	5 (0.3%)	1 (0.1%)	1 (0.1%)	0 (0%)	2 (0.1%)	0 (0%)	1 (0.2%)	2 (0.1%)	4 (0.1%)	28 (0.1%)	
	ROM	2 (0.1%)	4 (0.4%)	3 (0.2%)	0 (0%)	11 (0.7%)	3 (0.6%)	1 (0.2%)	6 (0.2%)	20 (0.4%)	171 (0.5%)	
Social groups	Disabled	4 (0.2%)	4 (0.4%)	5 (0.4%)	1 (0.2%)	4 (0.2%)	1 (0.2%)	3 (0.6%)	15 (0.5%)	12 (0.2%)	59 (0.2%)	0.002
	Displaced	14 (0.7%)	5 (0.6%)	4 (0.3%)	1 (0.2%)	8 (0.5%)	44 (8.5%)	4 (0.8%)	34 (1.2%)	100 (1.9%)	60 (0.2%)	<0.0001
	Immigrants	24 (1.3%)	5 (0.6%)	10 (0.8%)	6 (1.3%)	14 (0.9%)	5 (1%)	4 (0.8%)	20 (0.7%)	25 (0.5%)	70 (0.2%)	<0.0001
	Convicts	0 (0%)	4 (0.4%)	1 (0.1%)	1 (0.2%)	3 (0.2%)	0 (0%)	3 (0.6%)	9 (0.3%)	2 (0%)	27 (0.1%)	<0.0001
	Expectant mothers	15 (0.8%)	10 (1.1%)	11 (0.9%)	7 (1.5%)	8 (0.5%)	4 (0.8%)	2 (0.4%)	15 (0.5%)	51 (1%)	194 (0.5%)	<0.0001
	Children in state care	0 (0%)	0 (0%)	1 (0.1%)	0 (0%)	2 (0.1%)	0 (0%)	3 (0.6%)	9 (0.3%)	6 (0.1%)	19 (0.1%)	<0.0001
	Demobilized	0 (0%)	0 (0%)	0 (0%)	0 (0%)	3 (0.2%)	0 (0%)	3 (0.6%)	9 (0.3%)	4 (0.1%)	16 (0%)	<0.0001
	Victims of armed conflict	1 (0.1%)	4 (0.4%)	2 (0.2%)	0 (0%)	5 (0.3%)	6 (1.2%)	4 (0.8%)	9 (0.3%)	20 (0.4%)	33 (0.1%)	<0.0001

Where Bajo Cauca (BC), Magdalena Medio (MM), Nordeste (NE), Norte (NO), Occidente (OC), Oriente (OR), not georeferenced (NG), Suroeste (SO), Urabá (UR), and Valle de Aburrá (VA).

**Table 6:** Medical and symptomatically characteristics among patients, according to subregion location.

Variable		BC (n = 1874)	MM (n = 908)	NE (n = 1218)	NO (n = 475)	OC (n = 1644)	OR (n = 515)	NG (n = 491)	SO (n = 2741)	UR (n = 5196)	VA (n = 35,335)	*p*-Value
Medical consultation time (in days)		3 (3-4)	3 (3–3)	2 (2–3)	4 (3–4)	2 (2–3)	3 (3–4)	4 (3–4)	3 (3–3)	4 (4-4)	4 (4–4)	<0.0001
Hospitalized patients		907 (48.4%)	362 (39.9%)	457 (37.5%)	191 (40.2%)	426 (25.9%)	218 (42.3%)	99 (20.2%)	660 (24.1%)	3000 (57.7%)	8640 (24.5%)	<0.0001
Severe dengue		20 (1.1%)	11 1.2%)	17 (1.4%)	4 (0.8%)	13 (0.8%)	8 (1.6%)	3 (0.6%)	11 (0.4%)	66 (1.3%)	143 (0.4%)	<0.0001
Clinical deterioration time (in days)		4 (4–4)	4 (3–4)	4 (4–5)	5 (4–5)	4 (4–4)	4 (4–5)	5 (4–5)	4 (4–5)	4 (4–4)	5 (4–5)	<0.0001
Symptoms	Fever	1874 (100%)	908 (100%)	1218 (100%)	475 (100%)	1644 (100%)	515 (100%)	491 (100%)	2741 (100%)	5194 (99.9%)	35328 (99.9%)	<0.0001
	Headache	1651 (88.1%)	815 (89.8%)	964 (79.1%)	412 (86.7%)	1407 (85.6%)	425 (82.5%)	425 (86.6%)	2422 (88.4%)	4702 (90.5%)	30168 (85.4%)	<0.0001
	Retro-ocular pain	794 (42.4%)	482 (53.1%)	519 (42.6%)	241 (50.7%)	762 (46.4%)	239 (46.4%)	315 (64.2%)	1471 (53.7%)	2390 (46%)	17030 (48.2%)	<0.0001
	Myalgia	1558 (83.1%)	745 (82%)	1008 (82.8%)	419 (88.2%)	1430 (87%)	455 (88.3%)	442 (90%)	2384 (87%)	4331 (83.4%)	30595 (86.6%)	<0.0001
	Arthralgia	1354 (72.3%)	663 (73%)	876 (71.9%)	372 (78.3%)	1337 (81.3%)	398 (77.3%)	408 (83.1%)	2182 (79.6%)	3719 (71.6%)	27202 (77%)	<0.0001
	Rash	552 (29.5%)	327 (36%)	455 (37.4%)	201 (42.3%)	754 (45.9%)	246 (47.8%)	314 (64%)	1259 (45.9%)	1604 (30.9%)	18494 (52.3%)	<0.0001
	Abdominal pain	766 (40.9%)	347 (38.2%)	356 (29.2%)	94 (19.8%)	380 (23.1%)	161 (31.3%)	83 (16.9%)	653 (23.8%)	2231 (42.9%)	8166 (23.1%)	<0.0001
	Vomiting	624 (33.3%)	315 (34.7%)	301 (24.7%)	95 (20%)	336 (20.4%)	130 (25.2%)	92 (18.7%)	614 (22.4%)	2066 (39.8%)	7013 (19.8%)	<0.0001
	Diarrhea	256 (13.7%)	155 (17.1%)	165 (13.5%)	56 (11.8%)	202 (12.3%)	97 (18.8%)	57 (11.6%)	388 (14.2%)	1082 (20.8%)	5146 (14.6%)	<0.0001
	Drowsiness	109 (5.8%)	48 (5.3%)	58 (4.8%)	19 (4%)	39 (2.4%)	30 (5.8%)	13 (2.6%)	105 (3.8%)	309 (5.9%)	934 (2.6%)	<0.0001
	Hypotension	54 (2.9%)	18 (2%)	35 (2.9%)	13 (2.7%)	32 (1.9%)	13 (2.5%)	4 (0.8%)	53 (1.9%)	124 (2.4%)	477 (1.3%)	<0.0001
	Hepatomegaly	33 (1.8%)	13 (1.4%)	30 (2.5%)	8 (1.7%)	23 (1.4%)	15 (2.9%)	5 (1%)	54 (2%)	128 (2.5%)	310 (0.9%)	<0.0001
	Oral ecchymosis	87 (4.6%)	28 (3.1%)	47 (3.9%)	16 (3.4%)	67 (4.1%)	26 (5%)	9 (1.8%)	104 (3.8%)	133 (2.6%)	1361 (3.9%)	<0.0001
	Hypothermia	16 (0.9%)	3 (0.3%)	16 (1.3%)	0 (0%)	14 (0.9%)	5 (1%)	2 (0.4%)	19 (0.7%)	20 (0.4%)	137 (0.4%)	<0.0001
	Thrombocytopenia	764 (40.8%)	213 (23.5%)	323 (26.5%)	144 (30.3%)	340 (20.7%)	156 (30.3%)	61 (12.4%)	592 (21.6%)	1880 (36.2%)	6519 (18.4%)	<0.0001
	High hematocrit level	76 (4.1%)	27 (3%)	45 (3.7%)	29 (6.1%)	57 (3.5%)	39 (7.6%)	11 (2.2%)	145 (5.3%)	146 (2.8%)	1065 (3%)	<0.0001

Where Bajo Cauca (BC), Magdalena Medio (MM), Nordeste (NE), Norte (NO), Occidente (OC), Oriente (OR), not georeferenced (NG), Suroeste (SO), Urabá (UR), and Valle de Aburrá (VA).

**Table 7:** MW test *p*-values for the quantitative variables that rejected the null hypothesis of the KW test by subregions.

Subregion	BC	MM	NE	NO	OC	OR	NG	SO	UR
	Variable “age”								
MM	0.0001	-	-	-	-	-	-	-	-
NE	0.001	1	-	-	-	-	-	-	-
NO	<0.0001	<0.0001	<0.0001	-	-	-	-	-	-
OC	<0.0001	<0.0001	<0.0001	1	-	-	-	-	-
OR	<0.0001	<0.0001	<0.0001	1	0.52	-	-	-	-
NG	<0.0001	<0.0001	<0.0001	1	1	1	-	-	-
SO	<0.0001	<0.0001	<0.0001	1	0.52	0.007	0.47	-	-
UR	<0.0001	<0.0001	<0.0001	<0.0001	<0.0001	<0.0001	<0.0001	<0.0001	-
VA	<0.0001	<0.0001	<0.0001	1	1	0.66	1	0.0003	<0.0001
	Variable “medical consultation time”								
MM	<0.0001	-	-	-	-	-	-	-	-
NE	<0.0001	0.002	-	-	-	-	-	-	-
NO	0.02	<0.0001	<0.0001	-	-	-	-	-	-
OC	<0.0001	0.04	1	<0.0001	-	-	-	-	-
OR	1	0.04	<0.0001	0.04	<0.0001	-	-	-	-
NG	0.11	<0.0001	<0.0001	1	<0.0001	0.11	-	-	-
SO	0.01	0.11	<0.0001	<0.0001	<0.0001	0.97	<0.0001	-	-
UR	<0.0001	<0.0001	<0.0001	1	<0.0001	<0.0001	1	<0.0001	-
VA	<0.0001	<0.0001	<0.0001	1	<0.0001	0.01	1	<0.0001	<0.0001
	Variable “clinical deterioration length time”								
MM	0.67	-	-	-	-	-	-	-	-
NE	0.57	0.01	-	-	-	-	-	-	-
NO	0.08	0.002	1	-	-	-	-	-	-
OC	1	0.29	1	0.87	-	-	-	-	-
OR	1	0.04	1	1	1	-	-	-	-
NG	0.05	0.003	1	1	0.41	1	-	-	-
SO	0.02	0.0001	1	1	1	1	1	-	-
UR	0.05	0.0002	1	1	1	1	0.74	1	-
VA	<0.0001	<0.0001	0.67	1	0.003	1	1	0.74	<0.0001

Where Bajo Cauca (BC), Magdalena Medio (MM), Nordeste (NE), Norte (NO), Occidente (OC), Oriente (OR), not georeferenced (NG), Suroeste (SO), Urabá (UR), and Valle de Aburrá (VA).

**Table 8:** Social, medical, and symptomatological characteristics among patients according to dengue type.

Variable		Dengue (n = 50,101)	Severe Dengue (n = 296)	*p*-Value
	Age	23 (20–25)	26 (26–26)	0.14
Age group	Early childhood (0–5)	3924 (7.8%)	29 (9.8%)	0.2
	Childhood (6–11)	5541 (11.1%)	40 (13.5%)	0.2
	Adolescence (12–18)	7477 (14.9%)	51 (17.2%)	0.3
	Early adulthood (19–26)	8359 (16.7%)	53 (17.9%)	0.6
	Adulthood (27–59)	20,669 (41.3%)	88 (29.7%)	<0.0001
	Old age (60+)	4131 (8.2%)	35 (11.8%)	0.03
Sex	Female	25,190 (50.3%)	149 (50.3%)	1
	Male	24,911 (49.7%)	147 (49.7%)	
Clinical variables	Medical consultation time (in days)	3 (3–4)	4 (4–5)	<0.0001
	Hospitalized patients	14,670 (29.3%)	290 (98%)	<0.0001
	Clinical deterioration time (in days)	4 (4–5)	5 (4-5)	0.27
Symptoms	Fever	50,092 (100%)	296 (100%)	1
	Headache	43,155 (86.1%)	236 (79.7%)	0.002
	Retro-ocular pain	24,099 (48.1%)	144 (48.6%)	0.9
	Myalgia	43,112 (86.1%)	255 (86.1%)	1
	Arthralgia	38,275 (76.4%)	236 (79.7%)	0.2
	Rash	24,092 (48.1%)	114 (38.5%)	0.001
	Abdominal pain	13,019 (26%)	218 (73.6%)	<0.0001
	Vomiting	11,425 (22.8%)	161 (54.4%)	<0.0001
	Diarrhea	7508 (15%)	96 (32.4%)	<0.0001
	Drowsiness	1599 (3.2%)	65 (22%)	<0.0001
	Hypotension	740 (1.5%)	83 (28%)	<0.0001
	Hepatomegaly	579 (1.2%)	40 (13.5%)	<0.0001
	Oral ecchymosis	1820 (3.6%)	58 (19.6%)	<0.0001
	Hypothermia	212 (0.4%)	20 (6.8%)	<0.0001
	Thrombocytopenia	10,776 (21.5%)	216 (73%)	<0.0001
	High hematocrit level	1570 (3.1%)	70 (23.6%)	<0.0001

**Table 9:** Results of the robust Cox regression for clinical deterioration time analysis using the extended Wald test *p*-value < 0.0001.

Variable	Coefficient	Exp (Coefficient)	SE	*p*-Value
Sex (male)	0.047	1.048	0.019	0.013
Type of dengue (severe)	−0.104	0.902	0.070	0.139
Type of settlement (populated center)	0.120	1.127	0.037	0.001
Type of settlement (rural–dispersed)	−0.010	0.990	0.032	0.760
Subregion (MM)	0.154	1.166	0.073	0.036
Subregion (NE)	−0.153	0.858	0.067	0.022
Subregion (NO)	−0.192	0.826	0.093	0.039
Subregion (OC)	0.013	1.014	0.068	0.843
Subregion (OR)	−0.129	0.879	0.087	0.137
Subregion (SO)	−0.073	0.930	0.059	0.215
Subregion (UR)	−0.164	0.848	0.044	<0.0001
Subregion (VA)	−0.156	0.856	0.041	<0.0001

Where standard error (SE), Magdalena Medio (MM), Nordeste (NE), Norte (NO), Occidente (OC), Oriente (OR), Suroeste (SO), Urabá (UR), and Valle de Aburrá (VA).

**Table 10:** Distribution of the population corresponding to the demographic groups in each subregion: 46% and 64% of the urban and rural population in CB are in poverty, respectively. However, because each settlement has a different population size, the percentage of the total does not sum to 100%. Source: Departamento Administrativo Nacional de Estadística (DANE, Spanish acronym), www.dane.gov.co (accessed on 31 January 2022).

Subregion	BC	MM	NE	NO	OC	OR	SO	UR	VA
Poverty									
Urban	46%	28%	27%	24%	24%	17%	24%	40%	10%
Rural	67%	48%	56%	53%	52%	36%	47%	71%	22%
Total	56%	35%	42%	41%	43%	31%	37%	59%	12%
Health barrier									
Urban	4%	4%	2%	3%	3 %	3%	3%	6%	3%
Rural	4%	3%	4%	4%	3 %	2%	3%	5%	3%
Total	4%	3%	4%	4%	3 %	3%	4%	5%	3%
No access to drinking water									
Urban	8%	2%	2%	2 %	1%	1%	1 %	5 %	1%
Rural	36%	23%	60%	60%	29%	37%	41%	70%	18%
Total	19%	12%	32%	26%	16%	14%	21%	43%	3%
Overcrowding									
Urban	19%	10%	8%	7%	9%	6%	6%	15%	4%
Rural	15%	6%	6%	6%	7%	4%	4%	14%	3%
Total	17%	7%	6%	7 %	7%	5%	5%	15%	4%

Where Bajo Cauca (BC), Magdalena Medio (MM), Nordeste (NE), Norte (NO), Occidente (OC), Oriente (OR), Suroeste (SO), Urabá (UR), and Valle de Aburrá (VA).

## Data Availability

Not applicable.
